# Presence of periodontitis may synergistically contribute to cancer progression via Treg and IL-6

**DOI:** 10.1038/s41598-022-15690-w

**Published:** 2022-07-08

**Authors:** Ryo Kajihara, Hironori Sakai, Yibing Han, Kei Amari, Makiko Kawamoto, Yusuke Hakoyama, Sachiho Nagashio, Shin-ichi Yamada, Hideki Sanjo, Hiroshi Kurita

**Affiliations:** 1 Department of Dentistry and Oral Surgery, Shinshu University School of Medicine, 3-1-1, Asahi, Matsumoto, 390-8621 Japan; 2 Department of Molecular and Cellular Immunology, Shinshu University School of Medicine, Matsumoto, Japan

**Keywords:** Medical research, Oncology

## Abstract

A close causal relationship has been suggested to exist between cancer and periodontitis. We hypothesized that the immune surveillance system is impaired in patients with periodontitis, which contributes to cancer development and growth. Therefore, the present study investigated the relationship between immune surveillance mechanisms and periodontitis in cancer patients. The presence or absence of periodontitis was assessed and the peripheral blood (PB) concentrations of IL-6, immunosuppressive cytokines (VEGF, TGF-β1, and CCL22) and proportion of T regulatory cells (Treg, CD3 + CD4 + CD25 + Foxp3 +) were measured. Subjects were classified into the following four groups: non-cancer patients without periodontitis (C − P −), non-cancer patients with periodontitis (C − P +), cancer patients without periodontitis (C + P −), and cancer patients with periodontitis (C + P +). The results of a multivariate analysis showed that the PB concentration of IL-6 was significantly higher in C + than in C- and higher in C + P + than in C + P −. The PB proportion of Treg was significantly higher in C + P + than in C + P −, C − P + , and C − P −. The results of this study suggested that the presence of periodontitis and cancer synergistically increased Treg in PB, which may be one of the underlying causes of immunosuppression and immune evasion in cancer. It was also suggested that the presence of periodontal disease and/or cancer also increases IL-6 in PB, which would be associated with cancer progression. These results suggest the possibility that the presence of periodontitis might synergistically contribute to cancer progression.

## Introduction

Cancer immunity has been attracting increasing attention in recent years. In the process of cancer development and proliferation, cancer evades immune surveillance via a number of mechanisms. Two mechanisms have been confirmed: immunosuppression triggered by genetic aberrations in cancer cells and that by antitumor T cells that have already been induced^1,2^. In the former, genetic abnormalities in cancer cells induce interleukin-6 (IL-6) and immunosuppressive cytokines, such as transforming growth factor-β (TGF-β), interleukin-10 (IL-10), vascular endothelial growth factor (VEGF), and macrophage-derived chemokine (CCL22), as well as immunosuppressive cells [regulatory T cells (Treg) and bone marrow-derived immunosuppressive cells], which suppress the function of dendritic cells and antitumor T cells^3^.

The relationship between periodontal disease and cancer has been the focus of research based on findings showing that the risk of cancer development and cancer mortality are higher in patients with periodontal disease^4–12^. We also reported that the prevalence of severe periodontitis was higher in cancer patients^13^. However, the mechanisms contributing to this relationship remain unclear. A recent study showed that the number of Treg cells in peripheral blood (PB) was significantly higher in patients with periodontal disease than in those without periodontal disease^14^. Periodontitis, an inflammatory disease, is caused by oral microbiota, including *P. gingivalis*, T*reponema denticola*, and *Tannerella forsythia*^15^. Gingipain proteases produced by *P. gingivalis* affect the integrity of the cytokines IL-6 which are produced in response to the infection^16^.

Periodontitis develops and progresses as a result of an interaction between the immune system of the host and periodontal pathogens. Cytokines and immune cells play a crucial role in the modulation of host–pathogen homeostasis^17^. Treg are a key immunoregulatory element that has an anergic phenotype and plays an important role in the suppression of exacerbated immune responses, thereby maintaining homeostasis, which also preserves physiological/functional tissue homeostasis^14,18^. Present periodontitis affected systemic disease such as diabetes, atherosclerosis. Several previous studies indicated that higher prevalence of periodontitis in diabetic patients, and IL-6 might mediate periodontitis in diabetes^19,20^. And others studies showed that *A. actinomycetemcomitans*, *P. gingivalis*, *T. forsythia*, *T. denticola*, and *F. nucleatumare* related to higher risk of atherosclerosis, and some cardiovascular diseases are related to chronic inflammation^21^. Therefore, the presence of chronic inflammation, such as periodontitis, in the body may reduce systemic immune surveillance functions (e.g., increases in Treg), which may, in turn, promote the development and progression of cancer.

Based on these findings, we hypothesized that the immune surveillance system is impaired in patients with periodontitis, which contributes to cancer development and growth. Therefore, the present study investigated the relationship between immune surveillance mechanisms and periodontal disease in cancer patients.

## Materials and methods

The study protocol was approved by the Ethics Committee of Shinshu University School of Medicine (Approval number #4144). The present study was performed in accordance with the Declaration of Helsinki (2013 Fortaleza revision) and Ethical Guidelines for Medical Research for Humans.

### Patients

Subjects were recruited for the present study from patients who attended the Oral Management Center of Shinshu University Hospital and the Oral Disease Center of Aizawa Hospital between November 2018 and March 2020. Inclusion and exclusion criteria are summarized in Table 1. Data that may influence immunosuppression, cancer, or periodontitis (age, sex, smoking status (the Brinkman index), diabetes mellitus (DM), and number of teeth) were retrieved from patient medical records.

**Table 1:** Inclusion and exclusion criteria.

**Inclusion criteria**	
Cancer patients	20 years of age or older, diagnosed with solid cancer, and not yet started treatment
Non-cancer patients	20 years of age or older without any history of cancer
**Exclusion criteria**	
Any previous treatment for malignancy	
Edentulous	
Immunosuppressive disease	
Using drugs with immunosuppressive effects	
Recent active treatment for periodontitis	

### Assessment of periodontitis

We conducted an oral examination and periodontal disease assessment according to the Standard Adult Dental Examination Program/Health Guidance Manual (Japan Dental Association) and Periodontal disease screening program 2015 (Ministry of Health, Labour and Welfare, https://www.mhlw.go.jp/file/06-Seisakujouhou-10900000-Kenkoukyoku/manual2015.pdf) including a radiological examination using dental panoramic radiographs. Patients a probing depth < 3 mm, no bleeding on probing, and no radiographic evidence of horizontal alveolar bone loss were classified did not have periodontitis, while those with the opposite findings had periodontitis. Diagnosis of periodontal disease by clinical and imaging examination was performed by experienced dentists with prior training.

### PB sample collection and component analysis

PB was collected from the antecubital vein into EDTA-coated vacutainer tubes and thrombin-coated vacutainer tubes. Peripheral blood mononuclear cells (PBMC) obtained from the former were used for a flow cytometry (FCM) analysis and serum from the latter to analysis IL-6, VEGF, TGF-β1, and CCL22 concentrations measured by SRL Ltd. (Tokyo, Japan) and LSI Medience Corporation Ltd. (Tokyo, Japan).

### FCM analysis

Foxp3 is a specific marker of Treg. PBMC were separated from collected blood samples and Treg were detected by the expression of CD25 and Foxp3. We used the following fluorochrome-conjugated antibodies to stain the cell surface antigens: PerCp/Cy5.5 anti-human CD3, Brilliant Violet 421 anti-human CD4, APC anti-human CD25 (BioLegend, San Diego, CA, USA), and Ghost Rad780 Viability dye (Tonbo Bioscience, San Diego, CA, USA). We permeabilized cells using a Human Foxp3 buffer set kit (BD Bioscience, San Diego, CA USA), and stained Foxp3 using PE anti-human Foxp3 (BioLegend, San Diego, CA, USA). FCM was performed using BD FACS CantoII(Ver1.1, Diva6.1, BD Bioscience, San Diego, CA USA) and BD FACSDiva9, FlowJo (BD Bioscience, San Diego, CA USA) was used for data analysis. The gating strategy for the isolation of Treg involved the following steps: (1) isolation of live cells, (2) gating to isolate CD3- and CD4-positive cells, and (3) isolation of CD25- and Foxp3-positive cells (Supplementary Fig. 1).

### Statistical analysis

Statistical analyses, including Pearson’s test, the Wilcoxon test, Pearson’s correlation coefficient, Spearman’s correlation coefficient by rank, Tukey’s HSD test, the Steel–Dwass test, and a multivariate regression analysis, were performed using PC software (JMP v13.2, SAS Inc., NC, USA). All *p*-values < 0.5 were considered to indicate statistical significance.

## Results

Thirty cancer and 31 non-cancer patients agreed with informed consent to participate in the present study. Tumor sites in cancer patients were the head and neck (12 patients), lungs (5), colon (4), pancreas (3), breast (2), esophagus (2), duodenum (1), and bile duct (1). Among the 61 patients enrolled in the present study, 42 were diagnosed with periodontitis. Patients were classified into the following four groups: non-cancer patients without periodontitis (C − P −, 14 patients), non-cancer patients with periodontitis (C − P + , 17), cancer patients without periodontitis (C + P −, 5), and cancer patients with periodontitis (C + P + , 25). The characteristics of each group are shown in Table 2. Significant differences were observed in age, the Brinkman index, and the number of teeth among the groups. Mean age was lower in the C − P − group than in the other groups (*p* < 0.01). The Brinkman index was higher in the C + P + group than in the C − groups (*p* < 0.05). The number of teeth was significantly lower in the C + P + group than in the C − P − group (*p* < 0.01).

**Table 2:** Patient characteristics.

		C − P − (*n* = 14)	C − P + (n = 17)	C + P − (*n* = 5)	C + P + (n = 25)	*p*-value	
Sex	Female (21)	5	4	4	8	NS	Pearson’s test
	Male (40)	9	13	1	17		
Age	Mean ± SE	41.4 ± 3.6*	59.6 ± 3.3	72.6 ± 6.0	69.5 ± 2.7	*p* < 0.01	Tukey’s HSD test
Diabetes mellitus	Present (4)	0	1	0	3	NS	Pearson’s test
	Absent (57)	14	16	5	22		
Brinkman index	Median (IQR)	0 (0–10)^†^	0 (0–106)†	0 (0–465)	320 (0–846)^†^	*p* < 0.05	Steel–Dwass test
Number of teeth	Mean ± SE	28.0 ± 1.4^ƒ^	24.2 ± 1.3	21.4 ± 2.4	22.8 ± 1.1^ƒ^	*p* < 0.01	Tukey’s HSD test

C + : With cancer, C −: Without cancer.

P + : With periodontitis, P −: Without periodontitis.

*Significant difference between C −/P − and the others (Tukey’s HSD test, *p* < 0.01).

^†^Significant difference between C + /P + and either C − /P − or C − /P + (the Steel–Dwass test, *p* < 0.05).

^ƒ^Significant difference between C + /P + and either C − /P − (Tukey’s HSD test, *p* < 0.01).

**Figure 1:**
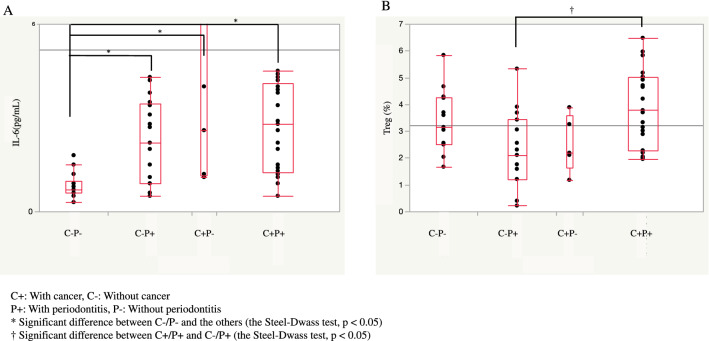
Comparison of IL-6 concentrations and the proportion of Treg in the peripheral blood of patients with or without cancer/periodontitis (Box-and-whisker diagram created by JMP v13.2, SAS Inc., NC, USA). (**A**) The concentration of IL-6 (pg/mL) was significantly lower in patients with neither cancer nor periodontitis (C − P −) than in those with cancer and/or periodontitis (C − P +, C + P −, and C + P +). (**B**) The proportion of Treg was significantly higher in patients with both cancer and periodontitis (C + P +) than in non-cancer patients without periodontitis (C − P +).

**Figure 2:**
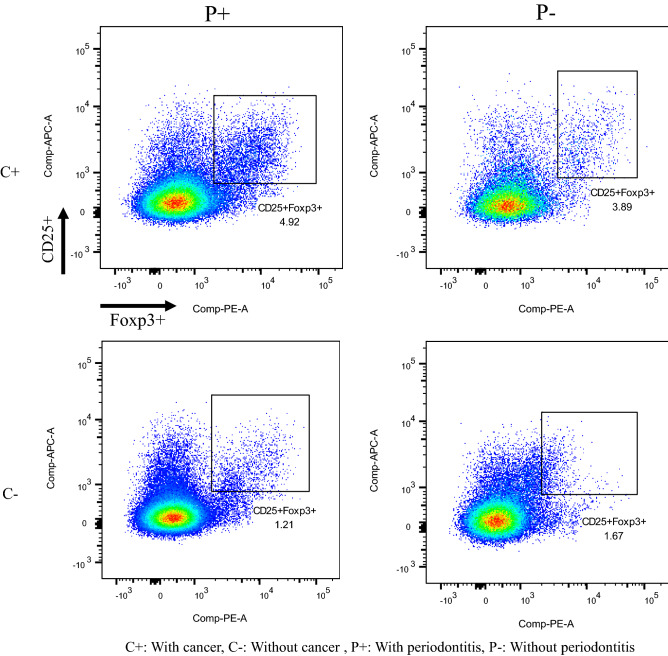
An example of a flow cytometric analysis (pseudo color plot) of the Treg proportion of CD3 + CD4 + lymphocytes in each group.

The results of a univariate analysis of the relationship between the levels of IL-6, immunosuppressive molecules or proportion of Treg and influencing factors (sex, age, the Brinkman index, DM, number of teeth, presence/absence of cancer, and periodontitis) are summarized in Table 3. No correlations were observed between TGF-β1, VEGF, or CCL22 concentrations and sex, the presence/absence of DM, age, the Brinkman index, or the number of teeth. Furthermore, no correlations were noted between TGF-β1, VEGF, or CCL22 concentrations and the presence/absence of cancer and/or periodontitis. On the other hand, correlations were detected between the concentration of IL-6 and either the presence/absence of DM or cancer/periodontitis. IL-6 concentrations were significantly higher in DM patients than in non-DM patients (median 4.3 vs. 1.5 pg/mL, *P* < 0.01), and were lower in C − P − patients than in the three other groups (median 0.7 vs. 2.2, 2.6, and 2.8 pg/mL, *P* < 0.05; Fig. 1A). In addition, a correlation was observed between the proportion of Treg and cancer/periodontitis. The proportion of Treg was significantly higher in C + P + patients than in C − P + patients (median 3.78 vs. 2.11%, *p* < 0.05; Figs. 1B and 2). And the proportion of Treg in PB was slightly higher in cancer patients than in non-cancer patients [median (IQR); 3.515% (2.1925–4.9875) vs. 2.56% (1.83–3.6975), *p* = 0.052]. However, no significant differences were observed in PB Treg between patients with and without periodontitis [median (IQR); 3.105% (2.0875–4.7625) vs. 3.16% (2.17–3.9775), *p* = 0.826].

**Table 3:** Results of the univariate analysis.

		IL-6 (pg/mL)			TGF-ß1 (ng/mL)			VEGF (pg/mL)			CCL22 (pg/mL)			Treg (%)		
		Median IQR			Median IQR			Median IQR			Median IQR			Median IQR		
Sex	Female (21)	1.4	1–3.4	*p* = 0.95	18.3	15.15–24.4	*p* = 0.095	229	143.5–418	*p* = 0.80	643	506.5–727	*p* = 0.56	3.15	2.15–3.795	*p* = 0.83
	Male (40)	1.75	0.825–3.725		21.5	17.725–26.7		248	151–483.25		634	563–792		3.06	2.05–4.65	
Diabetes mellitus	Presence (4)	4.3	4.05–4.475	*p* < 0.01	19.7	16.25–24.875	*p* = 0.73	222	63–368.5	*p* = 0.68	739.5	403.25–984.25	*p* = 0.59	3.15	2.1–5.01	*p* = 0.84
	Absence (57)	1.5	0.85–3		20.5	17.15–26.1		246	150–458.5		637	541.5–762.5		3.06	2.11–4.265	
Cancer/ Periodontitis	C − P − (14)	0.7*	0.6–0.975	*p* < 0.05	24.2	19.275–27.825	NS	250.5	169.75–314.75	NS	643	521.5–802.5	NS	3.15	2.515–4.265	*p* < 0.05
	C − P + (17)	2.2	0.9–3.45		20.5	27–26.3		296	150.5–556.5		641	587–783		2.11†	1.21–3.43	
	C + P − (5)	2.6	1.15–80.5		17.3	13.85–22.6		192	105.5–579		493	372.5–565.5		2.19	1.645–3.575	
	C + P + (25)	2.8	1.25–4.1		19.8	13.08–25.3		207	121.5–467		543	546.5–809.5		3.78†	2.28–5.01	

	IL-6 (pg/mL)		TGF-ß1 (ng/mL)		VEGF (pg/mL)		CCL22 (pg/mL)		Treg (%)							
	Correlation coefficient		Correlation coefficient		Correlation coefficient		Correlation coefficient		Correlation coefficient							
Age	*r* = 0.194	NS	*r* = 0.330	NS	*r* = 0.291	NS	*r* = 0.397	NS	r = − 0.102	NS						
Brinkman index	*r* = − 0.034	NS	*r* = 0.620	NS	*r* = 0.534	NS	*r* = 0.142	NS	*r* = 0.173	NS						
Number of teeth	*r* = 0.094	NS	*r* = 0.641	NS	*r* = 0.506	NS	*r* = 0.876	NS	*r* = − 0.024	NS						

C + : With cancer, C −: Without cancer.

P + : With periodontitis, P −: Without periodontitis.

*Significant difference between C − P − and the others (the Steel–Dwass test, *p* < 0.05).

^†^Significant difference between C + P + and C − P + (the Steel–Dwass test, *p* < 0.05).

The result of multivariate analysis is shown in Table 4. The relationships between either IL-6 concentrations or the proportion of Treg and influencing factors were analyzed. Regarding IL-6, the presence of cancer and the presence of periodontitis in cancer patients had a significant impact on the PB concentration of IL-6 (C − vs. C + , estimate − 7.534, p < 0.05 and C + P + vs C + P −, estimate 14.903, *p* < 0.01). Concerning Treg, the presence of both cancer and periodontitis had a significant impact on the PB proportion of Treg (C − and C + P − vs. C + P + , estimate − 0.774, *p* < 0.01). Age also had a significant impact on the proportion of Treg (estimate − 0.033, *p* < 0.05). These results of the multivariate analysis adjusted for confounding factors showed that the presence of cancer and/or periodontitis was associated with a higher PB concentration of IL-6 and proportion of Treg.

**Table 4:** Results of the multivariate analysis (multivariate regression analysis).

	Estimate	95%CI	*t*-value	*p* value	VIF
**A-IL6**					
Sex (female/male)	0.804	-5.574–7.182	0.250	0.801	1.489
Age	0.257	− 0.113–0.627	1.390	0.170	1.716
Brinkman index	− 0.002	− 0.019–0.016	− 0.200	0.840	1.993
Diabetes Mellitus (with/without)	2.077	− 8.874–13.028	0.380	0.705	1.248
Number of teeth	0.972	− 0.034–1.978	1.940	0.058	1.335
C − /C +	− 7.534	− 14.739–0.328	− 2.100	0.041	2.101
C + P + /C + P −	14.903	5.057–24.749	3.040	0.004	1.517
**B. Treg**					
Sex (female/male)	0.102	− 0.355–0.559	0.450	0.656	1.370
Age	-0.033	-0.060 –0.006	− 2.440	0.019	1.614
Brinkman index	0.000	− 0.001–0.001	0.160	0.871	1.655
Diabetes Mellitus (with/without)	0.097	− 0.756–0.950	0.230	0.820	1.074
Number of teeth	− 0.023	− 0.100–0.055	− 0.590	0.559	1.292
C − P − , C − P + , C + P − /C + P +	− 0.774	− 1.235 to − 0.313	− 3.380	0.002	1.420

C + : With cancer, C −: Without cancer.

P + : With periodontitis, P −: Without periodontitis.

CI: Confidential Interval, VIF: Variance Inflation Factor.

## Discussion

The present study is the first to examine the relationship between periodontitis and cancer from the aspect of immune surveillance mechanisms. We herein assessed the proportion of immunosuppressive cells and levels of IL-6 and immunosuppressive cytokines in the PB of patients with periodontal disease and/or cancer. Patients were classified into the four groups (C − P −, C − P + , C + P −, and C + P +) for comparison.

In the present study, PB levels of IL-6 and the proportion of Treg correlated with the presence and absence of cancer and/or periodontitis in both the univariate and multivariate analyses. Age and the smoking status significantly differed among the four groups compared in the present study. This was expected because the incidence of cancer and periodontal disease increases with age. Since age and the smoking status are considered to affect the immune status of the host^22,23^, adjustments for these confounding factors were warranted. Differences in the number of remaining teeth were also observed, but were naturally expected because periodontal disease is the most common reason for tooth loss in middle-aged and older individuals. Differences in the concentrations of pro-inflammatory cytokines according to the number of teeth affected by periodontal disease also need to be considered and their influence warrants further study. The results of the multivariate analysis adjusted for confounding factors showed that the presence of cancer and/or periodontitis was associated with a higher PB concentration of IL-6 and proportion of Treg, confirming the influence of the presence of cancer and periodontitis on the immune surveillance system of the host.

In this study the number of cancer patients without periodontitis (5 patients in C + P −) was smaller than in the other groups. Cancer and periodontal disease are both common in middle-aged and older individuals, and the prevalence of periodontal disease is higher in cancer patients^13^. We considered this imbalance in the number of patients to be unavoidable and proceeded with analyses. However, in the results of this study, the C + P + group had a significantly higher percentage of Tregs than the other three groups. This result suggests the existence of a synergistic effect between periodontal disease and cancer.

The concentration of IL-6 was significantly higher in patients with cancer and/or periodontitis than in those with neither cancer nor periodontitis. Previous studies suggested that IL-6 was associated with periodontitis. Salivary concentrations of IL-6 were shown to be higher in chronic periodontitis patients than in healthy controls^24,25^. Pro-inflammatory and anti-inflammatory cytokines have been detected in periodontal lesions, gingival crevice fluid, and gingival cells^26^. Furthermore, high expression levels of proinflammatory cytokines, including IL-6, were found in periodontitis and triggered osteoclast activity, which caused bone resorption^27^. IL-6 is also one of the major cytokines in cancer and plays a key role in cancer progression, metastasis, and therapeutic resistance^28^. It exerts direct stimulatory effects on many cancer cells through several signal pathways^29,30^. The present results revealed that IL-6 concentrations were increased in PB, but not in the cancer/periodontitis microenvironment. Higher levels of cytokines, including IL-6, in PB have been reported in patients with periodontitis^31^ and cancer^32^, which is consistent with the present results. The presence of local cancer or periodontal disease induced a higher PB level of IL-6, which may influence the general health of patients or may be associated with the development/progression of other diseases. We speculated that IL-6 from periodontitis may play a role in the development/progression of cancer. Cancer may be involved in the development/progression of periodontal disease and vice versa. IL-6 derived from gingival fibroblast induced VEGF through infiltrated macrophages in periodontitis site^33^. And TGF-β is also induced periodontitis site and play a role in wound healing and immunosuppression action^34^. In cancer site, overproduction of TGF-β induce immunosuppression in tumor and in lymph nodes, through Treg and myeloid-derived suppressor cells induction^35^. And VEGF has a dual function in supporting tumor progression, by inducing vessel formation and by acting as an immunosuppressive factor^36^. However, in this study, concentration of VEGF and TGF-β1 in PB was not significant differences between each group. We need to further study correlation between cancer and periodontitis regarding immunosuppressive cytokines.

The proportion of Treg in PB was significantly higher in patients with both cancer and periodontitis than in those without cancer or in cancer patients without periodontitis. This result suggests that the presence of periodontitis in cancer patients resulted in an additional increase in the proportion of Treg in PB. To the best of our knowledge, this is the first study to report a synergistic relationship between periodontitis and cancer in Treg-mediated immunosuppression.

CD4^+^CD25^+^Treg expressing the transcriptional factor Foxp3 are highly immunosuppressive and play central roles in the maintenance of self-tolerance and immune homeostasis^37,38^. Treg may suppress anticancer immunity, thereby hindering the protective immunosurveillance of neoplasia and hampering effective antitumor immune responses in tumor-bearing hosts, ultimately promoting tumor development and progression^39^. Elevated levels of Treg in the local microenvironment of cancer and a strong relationship between Treg and a poor prognosis in various types of cancer have been reported^40^. Furthermore, circulating Treg cells were shown to be elevated in several cancers and correlated with a poor prognosis^41,42^. In the present study, the proportion of Treg in PB was slightly higher in cancer patients than in non-cancer patients [median (IQR); 3.515% (2.1925–4.9875) vs. 2.56% (1.83–3.6975), *p* = 0.052].

Higher levels of Treg have been reported in the gingival tissues and PB of patients with chronic periodontitis^14,43,44^. Treg play a critical role in immunological tolerance and the suppression of inflammatory responses in the pathogenesis of periodontitis. In the present study, no significant differences were observed in PB Treg between patients with and without periodontitis [median (IQR); 3.105% (2.0875–4.7625) vs. 3.16% (2.17–3.9775), *p* = 0.826]. However, as noted above, a significant difference was observed between cancer patients with and without periodontitis. The presence of cancer and periodontal disease appears to synergistically increase Treg in PB. Previous studies reported that chronic and autoimmune diseases, such as rheumatoid arthritis (RA) and tuberculosis, had higher levels of Treg in the systemic circulation^45–47^. Patients with RA are at an increased risk of malignancies than the general population^48^. Periodontitis is a chronic inflammatory disease with compromised immunomodulatory mechanisms (immune tolerance). The systemic level of Treg is elevated with chronic or autoimmune disease, which may be one of the underlying causes of immunosuppression and immune evasion by cancer cells. These findings suggest that the presence of periodontitis contributes to immune evasion in cancer. In addition, the proportion of Treg was significantly lower with advancing age, which may reflect a decrease in the induction of Treg because of immune tolerance with aging^49^.

Previous studies demonstrated the importance of CCL22 in the migration of Treg^50,51^. In the present study, no significant differences were observed in the PB concentration of CCL22 in both cancer and/or periodontitis patients. Wertel et al. examined the PB, peritoneal fluid (PF), and tissue levels of CCL22 and Treg in patients with ovarian cancer, and reported that the percentage of Treg infiltrating tumors was significantly higher than in PF and PB, while no significant differences were noted in PB CCL22 concentrations between ovarian cancer patients and non-cancer patients^52^. CCL22 may be produced locally by tumor cells and myeloid dendritic cell^2^, and was also increased at periodontal inflammation sites^53,54^. These findings indicate that an increase of CCL22 in PB was not observed in patients with cancer or periodontitis because CCL22 increased locally and induced the migration of Treg.

In this study, we classified patients with a probing depth > 3 mm, bleeding on probing, and radiographic evidence of horizontal alveolar bone loss were classified with periodontitis. Our classification was different from the new EFP/AAP classification, and our classification might be insatisfaction. However, our result showed correlation presence destruction of periodontal tissue and cancer through Treg and IL-6 involved. And it was remained unclear, the duration of differences between periodontitis and cancer development, and immune response of the immune system concerned. Our result suggested that underlying periodontitis, no relevant the duration the time of development, might contribute cancer invasion through immune imbalance. Current study is the first report of correlation presence of periodontitis and cancer, but we need to further study to make clear presence of periodontitis contribution to cancer invasion.

In conclusion, we showed that the presence of periodontitis and cancer synergistically increased Treg in PB, which may be one of the underlying causes of immunosuppression and immune evasion in cancer. The presence of periodontal disease and/or cancer also increases IL-6 in PB, which would be associated with cancer progression. These results suggest the possibility that the presence of periodontitis might synergistically contribute to cancer progression.

## Supplementary Information

Supplementary Information 1.

Supplementary Information 2.

## Data Availability

The datasets used and/or analyzed during the current study are available from the corresponding author on reasonable request.
