# Supplementary material for: Presence of periodontitis may synergistically contribute to cancer progression via Treg and IL-6
Source: Sci Rep. 2022 Jul 8;12:11584. doi: 10.1038/s41598-022-15690-w (PMC9270385; doi:10.1038/s41598-022-15690-w)
Supplement: Supplementary file 1 — Supplementary Information 1. [file 41598_2022_15690_MOESM1_ESM.docx]

**Supplementary Figure 1.**

The gating strategy for the isolation of Treg involved the following steps: (A) isolation of lymphocytes (B) (C) isolation of single cells (D) isolation of live cells, (E) gating to isolate CD3- and CD4-positive cells, and (F) isolation of CD25- and Foxp3-positive cells.
