# Supplementary figures and images for: Presence of periodontitis may synergistically contribute to cancer progression via Treg and IL-6
Source: Sci Rep. 2022 Jul 8;12:11584. doi: 10.1038/s41598-022-15690-w (PMC9270385; doi:10.1038/s41598-022-15690-w)

## Slide 1
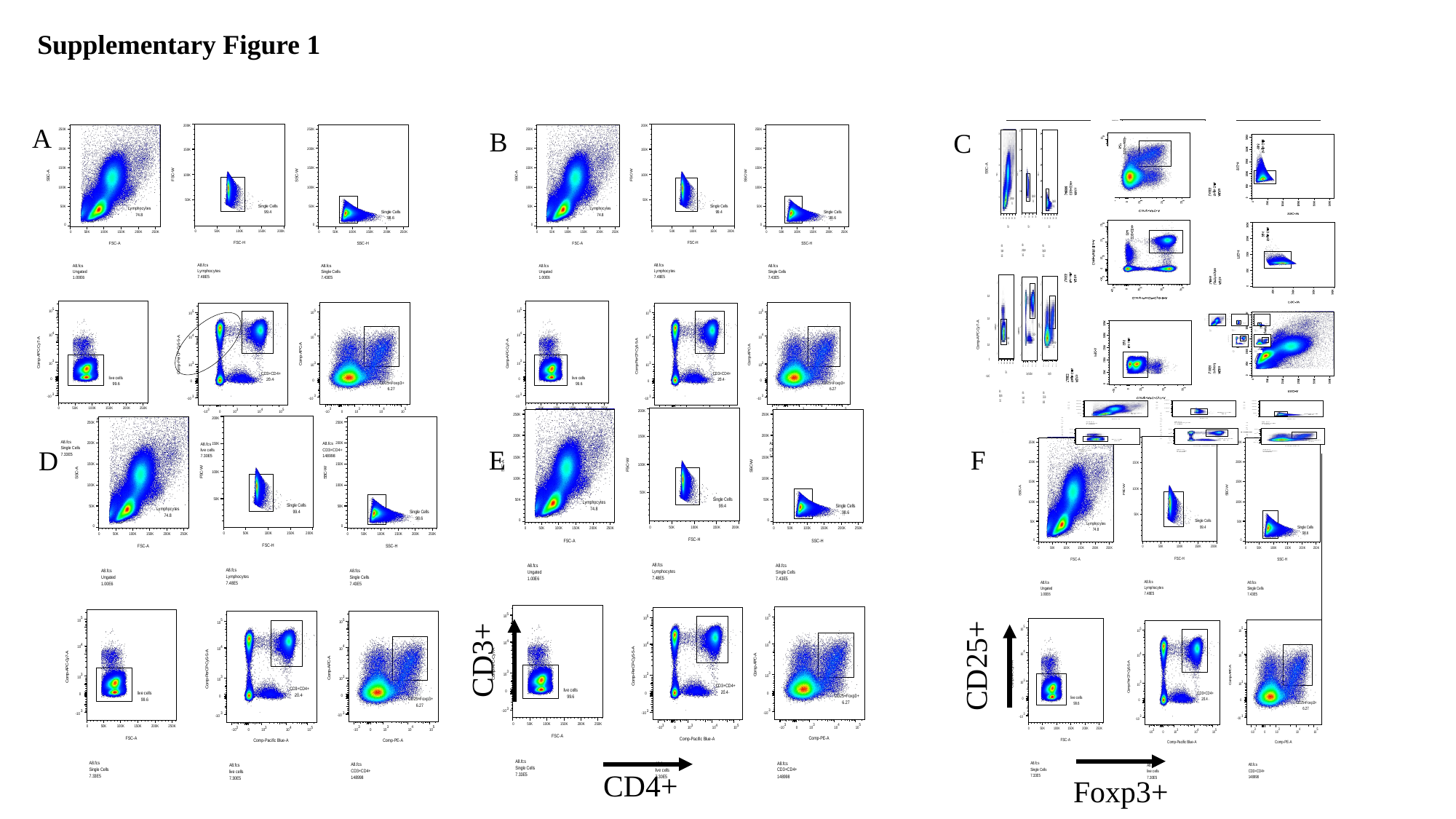

Supplementary Figure 1
A
CD3+
CD25+
CD4+
Foxp3+
B
C
F
E
D

Supplement: Supplementary file 2 — Supplementary Information 2. [file 41598_2022_15690_MOESM2_ESM.pptx]
